# Analysis of the Thermal Aging Kinetics of Tallow, Chicken Oil, Lard, and Sheep Oil

**DOI:** 10.3390/molecules29174191

**Published:** 2024-09-04

**Authors:** Yun-Chuan Hsieh, Hao Ouyang, Yulin Zhang, Donyau Chiang, Fuqian Yang, Hsin-Lung Chen, Sanboh Lee

**Affiliations:** 1 Department of Materials Science and Engineering, National Tsing Hua University, Hsinchu 300, Taiwan; ferrarihsieh@gmail.com (Y.-C.H.); houyang@mx.nthu.edu.tw (H.O.); 2 Chongqing Institute of Green and Intelligent Technology, Chinese Academy of Sciences, Chongqing 400714, China; yulin_zhang@foxmail.com; 3 National Applied Research Laboratories, Taiwan Instrument Research Institute, Hsinchu 300, Taiwan; dony@narlabs.org.tw; 4 Materials Program, Department of Chemical and Materials Engineering, University of Kentucky, Lexington, KY 40506, USA; fyang2@uky.edu; 5 Department of Chemical Engineering, National Tsing Hua University, Hsinchu 300, Taiwan; hlchen@che.nthu.edu.tw

**Keywords:** animal oils, UV-Vis spectrometry, electrical impedance, viscosity, acid titration

## Abstract

Understanding the thermal aging kinetics of animal oils is of vital importance in the storage and applications of animal oils. In this work, we use four different techniques, including UV-Vis spectrometry, viscometry, impedance spectroscopy, and acid–base titration, to study the thermal aging kinetics of tallow, chicken oil, lard, and sheep oil in the temperature range from 120 °C to 180 °C. The evolutions of the UV-Vis absorbance, dynamic viscosity, electric impedance, and acid titration are discussed with the defect kinetics. The evolutions of the color centers, defects for dynamic viscosity, and electric dipoles follow second-order, first-order, and zero-order kinetics, respectively. The temperature dependence of rate constants for the evolutions of the UV-Vis absorbance, dynamic viscosity, electric impedance, and acid titration satisfies the Arrhenius equation with the same activation energy for individual animal oils. The activation energies are ~43.1, ~23.8, ~39.1, and ~37.5 kJ/mol for tallow, chicken oil, lard, and sheep oil, respectively. The thermal aging kinetics of the animal oils are attributed to the oxidation of triglycerides.

## 1. Introduction

Animal cooking oils have been closely related to our daily lives for a long history [1,2]. They play an important role in heat transfer during cooking and contribute to the taste and texture of cooked food [3]. Animal cooking oils can undergo microstructural changes at high temperatures and/or during cooking through chemical reactions, including hydrolysis, pyrolysis, oxidation, cyclization, and thermal polymerization due to oxygen and water vapor in the air [4,5,6]. This can lead to changes in the chemical and physical properties of animal cooking oils, reduce the storage life of animal cooking oils, and even directly affect the quality of final food [7]. Also, the chemical changes in animal cooking oils can introduce harmful content, such as aldehydes and alcohols, to human beings [8,9]. It is of practical importance to understand the effect of temperature on the physical and chemical properties of animal cooking oils.

Various techniques are available to evaluate the physical and chemical properties of cooking oils, including Fourier transform infrared (FTIR) spectroscopy, ultraviolet and visible (UV-Vis) spectroscopy, mass spectroscopy, viscometry, and electrical impedance spectroscopy. Chen et al. [10] discussed the evolution of polar compounds in edible oils and suggested that there are three processes of oxidation, hydrolysis, and polymerization, contributing to the formation of polar compounds. Kumar and Viswanathan [11] studied the UV transmission of chicken oil and reported ~85% of the absorption of UV radiation. They did not evaluate the temperature dependence of the absorption of the UV radiation of the chicken oil. Guillén and Cabo [7] analyzed the chemical composition of lard with FTIR spectroscopy and revealed the composition dependence of the frequencies of some bands. Talpur et al. [12] determined the peroxide value in canola oil with UV-Vis spectrometric analysis. Their results indicated the feasibility of UV-Vis spectrometry in measuring the peroxide value in edible oils. Lapcikova et al. [13] determined the thermal aging kinetics of olive, peanut, rapeseed, soybean, and sunflower oils from the temperature dependence of the UV-Vis spectra of the oils. They demonstrated that the heating process caused the increase in the oxidation products and the decrease in the chlorophyll. Dost and İdeli [14] determined polycyclic aromatic hydrocarbons in vegetable oils and barbecued food from the absorbance spectra and analyzed the recovery and reproducibility of the approach used in their work. Hassanien et al. [15] developed a nonlinear model to analyze the temperature dependence of viscosity for sunflower, cottonseed, and palm oils. Alviso et al. [16] demonstrated the increase in the viscosity of oil with increasing saturated acid. Zahir et al. [17] reported the dependence of the viscosity of oil on triglycerides and the molecular arrangement of fatty acids. Yadav et al. [18] found more saturated components in non-edible oil than in edible oil. Khaled et al. [19] reported the correlation among electric impedance, TPC, and viscosity for palm oil under heating. Yang et al. [20] observed that the increment of the dielectric constant and dielectric loss of soybean oil increased linearly with the frying time in the range of 0 to 30 h. However, there are few kinetic studies on the determination of the activation energies for the rate processes controlling the thermal aging kinetics of animal cooking oils [21,22].

Considering the wide use of animal cooking oils in our daily lives, we used different techniques to investigate the thermal aging kinetics of four different animal oils, including tallow, chicken oil, lard, and sheep oil. The temperature dependencies of the optical absorbances, viscosities, electric impedances, and FTIR spectra of the oils at high temperatures were characterized and analyzed. The primary objective of the present study is to elucidate the kinetics of thermal aging in animal oils, focusing specifically on the rate at which macroscopic properties, such as the optical properties, viscosity, and electrical impedance, change as a result of thermal aging. Our focus on the changes in macroscopic properties is crucial, because these properties directly impact the practical usability and shelf life of animal oils. By quantifying how these properties evolve over time, this study provides insights into the overall activation energy and the rate of thermal aging, which are essential for predicting and managing the stability of animal oils in real-world applications.

## 2. Results

**Figure 1:**
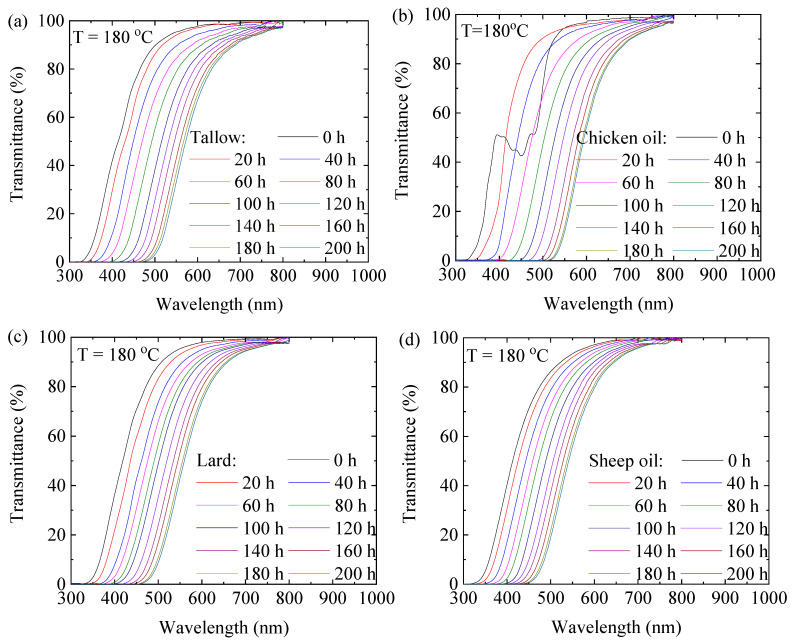
Transmittance spectra of the animal oils at 180 °C for different durations: (**a**) tallow, (**b**) chicken oil, (**c**) lard, and (**d**) sheep oil.

Figure 1 shows the transmittance spectra of the tallow, chicken oil, lard, and sheep oil at 180 °C in the wavelength range from 300 nm to 800 nm. The transmittance spectra of the tallow, chicken oil, lard, and sheep oil at 160 °C, 140 °C, and 120 °C are shown in Figures S1–S3 in the Supplementary Information. The transmittance for all the four animal oils decreases with the decrease in the wavelength and reaches 0% at the cutoff wavelength, and the transmittance spectrum undergoes a red shift with increases in the heating time. Such behavior is likely due to the changes in the chemical structures and/or chemical compositions, such as the formation and growth of color centers, during the heating process [23]. The largest red shift occurs at the highest temperature of 180 °C, revealing the temperature dependence of the changes in the chemical structures and/or chemical compositions.

According to Figure 1b and Figures S1b–S3b, the transmittance spectra of chicken oil exhibit fluctuations in the wavelength range from 400 nm to 500 nm at the onset of aging (i.e., 0 h). The substance responsible for the fluctuation signal is likely β-carotene, as it is known to show a strong absorption in the 400–500 nm range [24]. β-carotene, one of the most important carotenoids, cannot be synthesized by chickens themselves and is typically added to poultry feed as the primary source of vitamin A. Increasing the heating time at high temperatures causes the decomposition of the substance, as revealed by the transmittance spectra.

Using the transmittance spectra of the animal oils, we determined the difference between the transmittance at the onset of heating, *T_i_*_0_, and the corresponding one, *T_i_*_F_, at the end of heating (200 h). Figure S4 in the Supplementary Information shows the variation in (*T_i_* − *T_i_*_0_) with the wavelength for the heating process at different temperatures. It is interesting to note that maximum values of (*T_i_*_F_ − *T_i_*_0_) exist for individual animal oils at different temperatures. The corresponding wavelength increases with the increase in temperature.

The correlation between absorbance (A), reflectance (T_R_), and transmittance (T*_i_*) is described as follows:

(1) $$A+T_{i}+T_{R}=1$$

**Figure 2:**
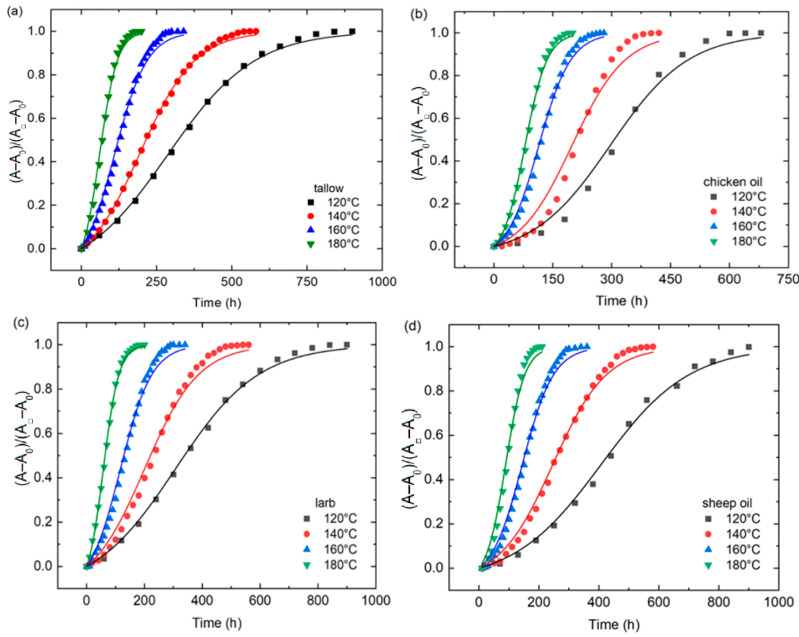
Temporal evolution of the absorbance differences (A − A_0_) of the animal oils at different temperatures at individual peak wavelengths: (**a**) tallow, (**b**) chicken oil, (**c**) lard, and (**d**) sheep oil.

Assuming T_R_ ≈ 0, the UV-Vis absorbances of the animal oils were calculated from the transmittance. Figure 2 shows the temporal evolution of the absorbance differences (A − A_0_) of the animal oils at corresponding temperatures at individual peak wavelengths. Here, A and A_0_ are the absorbances corresponding to the heating times of *t* and 0, respectively. Note that the absorbance was calculated as (1 − T*_i_*) instead of the Lambert–Beer law. It is evident that the absorbance at a given temperature increases with increases in the heating time and approaches plateaus for all the animal oils. Such a trend can be ascribed to the increase in the concentration of color centers with increased heating times, which lead to an increase in the absorbance. According to Figure 2, the absorbance is also an increasing function of the heating temperature at the same heating time, indicating the temperature dependence of the growth of color centers.

**Figure 3:**
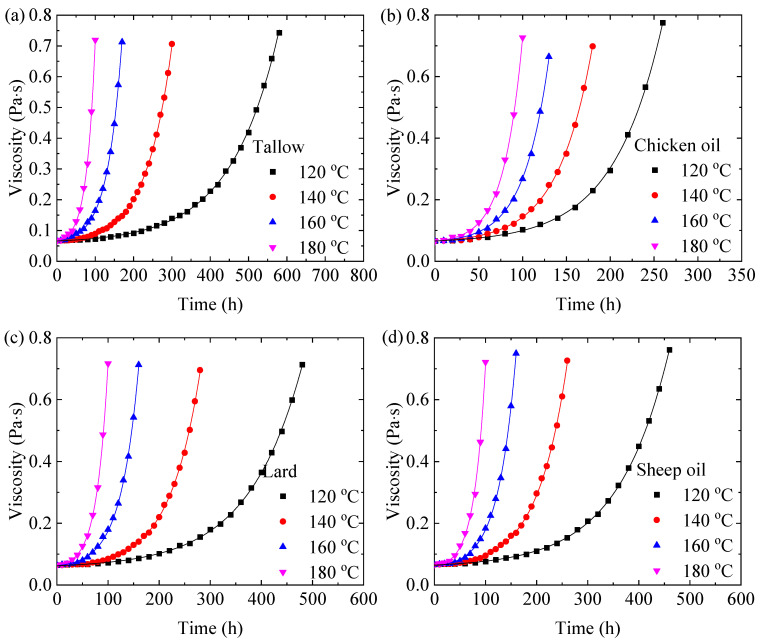
Temporal variations of the viscosities of the animal oils at different temperatures: (**a**) tallow, (**b**) chicken oil, (**c**) lard, and (**d**) sheep oil.

Figure 3 shows temporal variations in the dynamic viscosities of the animal oils at different temperatures. At the same heating temperature, the dynamic viscosity increases with the heating time for all the animal oils, indicating the presence of chemical reactions during the heating process, such as the formation of color centers. At the same heating time, the higher the heating temperature, the higher the dynamic viscosity. Such results reveal the dependence of chemical reactions on the heating temperature.

**Figure 4:**
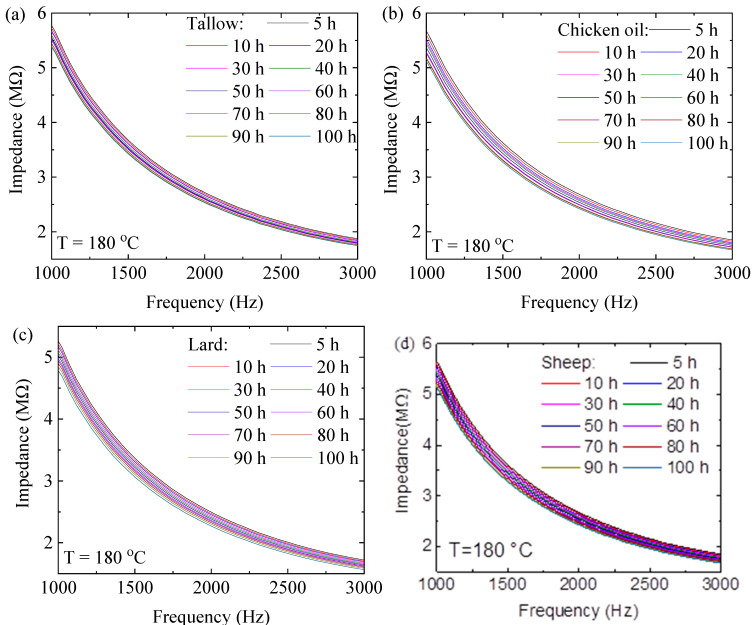
Frequency dependence of electric impedances of the animal oils at 180 °C at different heating times: (**a**) tallow, (**b**) chicken oil, (**c**) lard, and (**d**) sheep oil.

An LCR meter with an interdigital sensor of 100 µm of finger interspace was used to measure the variation in the electrical impedances of the animal oils in the frequency range from 1000 Hz to 3000 Hz. Figure 4 and Figures S5–S7 in the Supplementary Information show the frequency dependence of the electric impedances of the animal oils at different heating times at different temperatures. The electric impedance decreases nonlinearly with increases in the frequency under the same heating time at the same temperature for all the animal oils. At the same frequency and temperature, the electric impedance is a decreasing function of the heating time. Note that the electrical impedance of oils is a function of the fraction of the dipoles in the fat [25]. Increasing the fat content in an oil increases the fraction of dipoles and electric susceptibility. Thus, the decrease in the electric impedance is likely due to the increase in the dipoles with the increase in the heating time.

**Figure 5:**
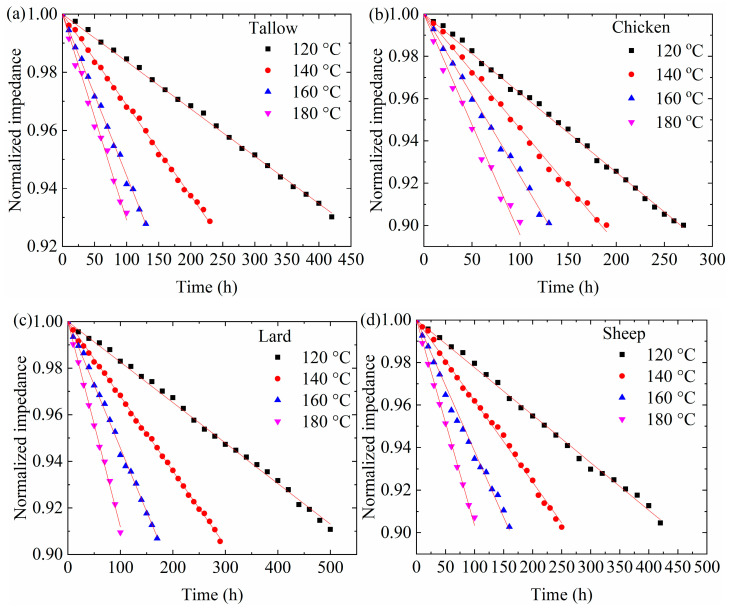
Temporal variation of normalized impedances of the animal oils at the frequency of 1000 Hz at different temperatures: (**a**) tallow, (**b**) chicken oil, (**c**) lard, and (**d**) sheep oil.

Using the results shown in Figure 4 and Figures S5–S7, we normalize the electric impedance at heating time *t* with the one at *t* = 0 h at the same temperature at the frequency of 1000 Hz. Figure 5 shows the temporal variation in the normalized impedance at different temperatures for the four different animal oils. Increasing the heating temperature causes the decrease in the normalized impedance. At the same heating time, the higher the heating temperature, the smaller the normalized impedance. Again, the results illustrate the dependence of the impedance on both the heating time and temperature.

**Figure 6:**
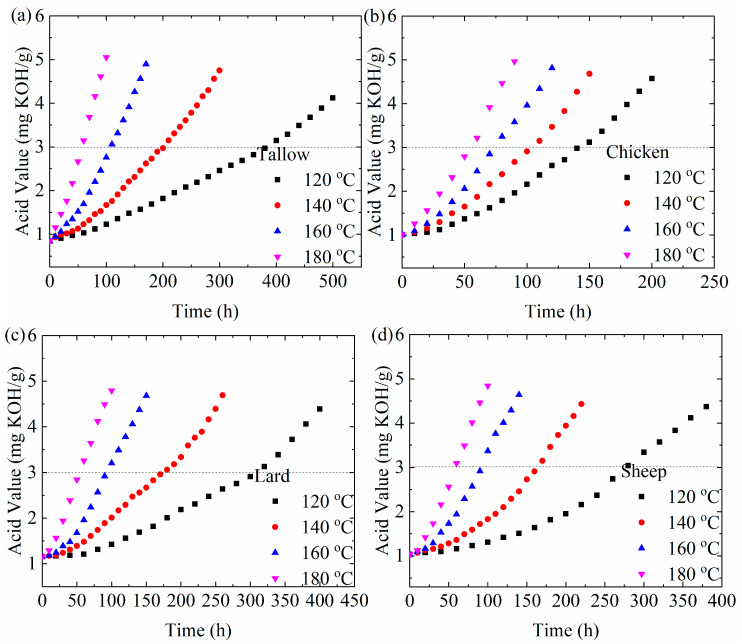
Temporal evolution of the acid values of the animal oils at different temperatures: (**a**) tallow, (**b**) chicken oil, (**c**) lard, and (**d**) sheep oil.

Acid-base titration provides a simple approach to analyze the deterioration of animal oils. In this work, we used KOH as a titrant. Figure 6 shows the temporal evolution of the acid values of the animal oils at different temperatures. It is evident that the acid value increases with the increases in the heating time at the same temperature and the heating temperature at the same heating time. Increasing the heating temperature and the heating time at high temperatures can cause the deterioration of the animal oils, as expected.

**Figure 7:**
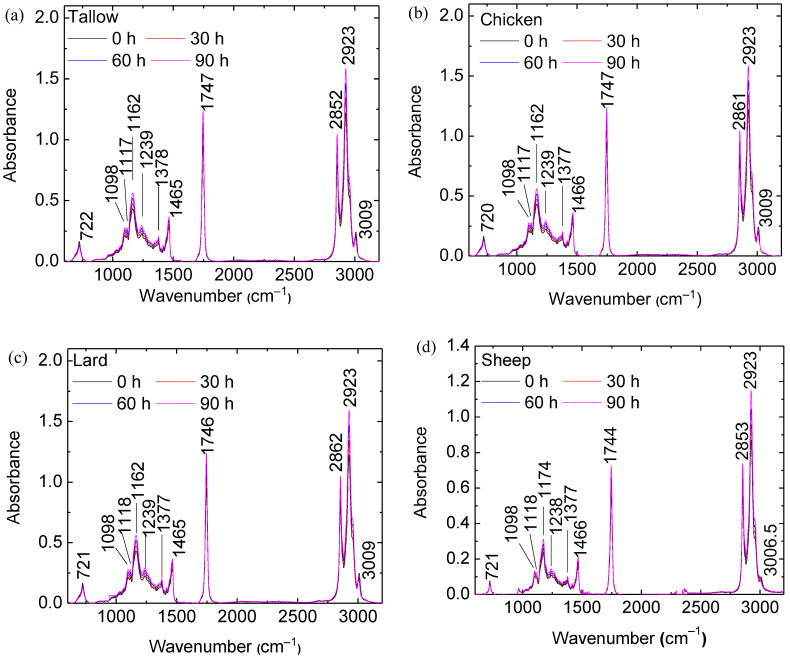
FTIR spectra of the animal oils for different heating times at 120 °C: (**a**) tallow, (**b**) chicken, (**c**) lard, and (**d**) sheep.

Animal oils with triglycerides as the main composition can undergo a cis-trans isomerization and produce isolated trans-fatty acids, which are suspected to cause arteriosclerosis and heart disease [24]. Also, animal oils can undergo oxidation and produce organic compounds including carboxylic acid and aldehyde [25]. Figure 7 shows the FTIR spectra of the animal oils for different heating times at 120 °C; enlarged views of the FTIR spectra are shown in Figures S8–S12 in the Supplementary Information. The FTIR spectra of the animal oils are highly similar. The main peak at 3010 cm^−1^ is associated with the C–H stretching vibration of a cis–double bond (–CH); the two sharp peaks at 2923 and 2852 cm^−1^ represent the symmetric and asymmetric stretching vibration of the aliphatic CH_2_ group, respectively. The sharp peak at 1747 cm^−1^ represents the ester carbonyl functional group of triglycerides; a peak at 1740 cm^−1^ represents the C=O stretching aldehyde. A peak at 1465 cm^−1^ is associated with the bending vibrations of the CH_2_ and CH_3_ aliphatic groups; a small peak at 1378 cm^−1^ represents the bending vibrations of the CH_2_ groups. The peaks at 1239, 1162, and 1098 cm^−1^ are related to the stretching vibration of the C–O ester groups. A peak at 1117 cm^−1^ represents aliphatic esters. A peak at 722 cm^−1^ is related to the CH_2_ rocking vibration and the out-of-plane vibration of cis-disubstituted olefins. All of the peaks are the same as the corresponding ones reported by Vlachos et al. [24].

The changes in peak intensities are related to the changes in the fractions of unsaturated and saturated fatty acids during the heating process. The decrease in the intensity peak at 1747 cm^−1^ is due to oxidation, and the decrease in the intensity peak at 722 cm^−1^ is related to the change of C=C cis to C=C trans bonds during the heating process. The oxidation produces other secondary products such as aldehydes, ketones, lower fatty acids, and hydrocarbons. Animal oils, which are heated at high temperatures for a long time, will become harmful to the human body. There is a slight shift in the peak wavenumber from 3007 cm^−1^ to 3004 cm^−1^, which is related to the change in the unsaturation degree of the animal oils. The heating contributes to the unsaturation of the oil.

## 3. Discussion

Tsai et al. [23] suggested that the evolution of color centers follows the second-order kinetic process; these authors obtained a closed-form expression of the temporal evolution of the concentration of color centers as follows:

(2) $$\frac{n_{A}-n_{A□}}{n_{A0}-n_{A□}}=1-\frac{1-e^{-\alpha_{A}t}}{1-{{\lambda \beta }_{A}e}^{-\alpha_{A}t}}$$

with

$$\lambda =\frac{n_{A□}-n_{A0}}{1-\beta_{A}(n_{A□}-n_{A0})}$$

Here, $n_{A}$ is the concentration of color centers at time *t*, $\alpha_{A}$ is the rate constant, $\beta_{A}$ is the proportional constant between the concentration of color centers and the absorbance ($A=\beta_{A}n_{A}$), $n_{A□}$ and n_A0_ are the concentrations of color centers at t=∞ and t=0, respectively. Using Equation (2) and $A=\beta_{A}n_{A}$, we have the following [23]:

(3) $$\frac{A-A_{0}}{A_{□}-A_{0}}=1-\frac{e^{-\alpha_{A}t}}{1-(A_{□}-A_{0})\left(1-e^{-\alpha_{A}t}\right)}$$

where $A_{0}=\beta_{A}n_{A0}$ is the absorbance at t=0, and $A_{□}=\beta_{A}n_{A□}$ is the absorbance at t=∞. Using Equation (3) to fit the data in Figure 2, we obtain the numerical values of the fitting parameter, $\alpha_{A}$, for the animal oils at different temperatures. For comparison, the fitting curves are included in the corresponding plots in Figure 2. It is evident that Equation (3) accurately describes the temporal evolution of the absorbance of the animal oils.

**Figure 8:**
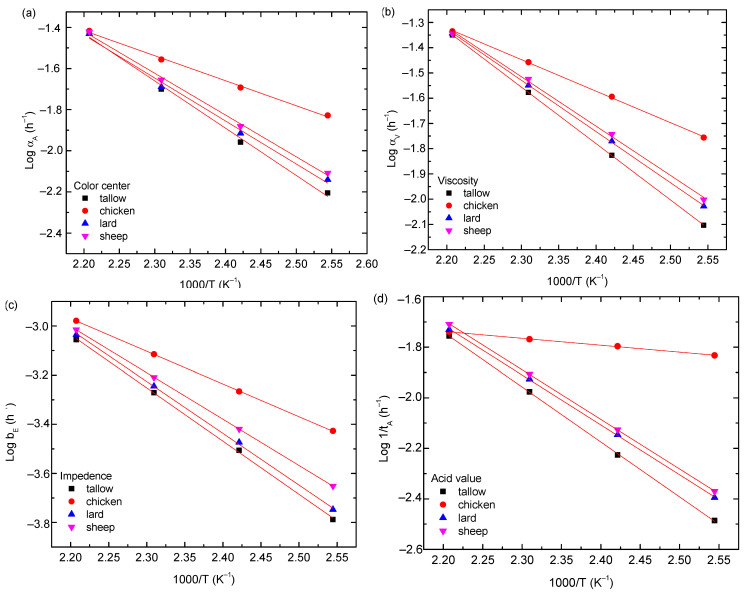
(**a**) Temperature dependence of $\alpha_{A}$ for the animal oils, (**b**) temperature dependence of $\alpha_{V}$ for the animal oils, (**c**) temperature dependence of b_E_ for the animal oils, and (**d**) temperature dependence of log(1/t_A_) of acid value for the animal oils.

It is known that the temperature dependence of the rate constant follows the Arrhenius relation. The temperature dependence of the rate constant is shown in Figure 8a for the animal oils, from which the activation energies for the evolution of color centers are found to be 43.8, 23.2, 39.8, and 38.8 kJ/mol for tallow, chicken oil, lard, and sheep oil, respectively. The livestock oils (tallow, lard, and sheep oil) have a greater activation energy of color centers than the poultry (chicken oil).

Assuming that the dynamic viscosity of the animal oils is proportional to the concentration of defects in the animal oils and the evolution of the defects for the dynamic viscosity follows the first-order kinetics, we have the following:

(4) $$\varphi =\varphi_{0}\exp\left(\alpha_{v}t\right)$$

where φ is the dynamic viscosity at time *t*, φ_0_ is the dynamic viscosity at *t* = 0, and α_V_ is the rate constant for the first-order kinetics. Using Equation (4) to fit the data in Figure 3, we obtain the numerical values of the fitting parameter, $\alpha_{V}$, for the animal oils at different temperatures. For comparison, the fitting curves are included in the corresponding plots in Figure 3. It is evident that Equation (4) accurately describes the temporal evolution of the dynamic viscosity of the animal oils.

As discussed above, the temperature dependence of the rate constant follows the Arrhenius relation. Figure 8b shows the variation in the rate constant with temperature for the animal oils, from which the activation energies for the evolution of defects are found to be 42.8, 23.4, 38.8, and 37.4 kJ/mol for tallow, chicken oil, lard, and sheep oil, respectively. Note that the activation energy represents the energy barrier to overcome the oxidation of triglycerides [26], implying that the smaller the activation energy, the easier the oxidation of triglycerides. Compared to the activation energies obtained from the absorbances, we note that the activation energies for the evolution of defects for the dynamic viscosity are basically the same as the corresponding ones obtained from the evolution of color centers. Such a result suggests that the oxidation of triglycerides is associated with the evolution of color centers. The livestock oils (tallow, lard, and sheep oil) have greater energy barriers for the oxidation of triglycerides than the poultry oil (chicken oil).

The thermal aging kinetics of animal oils can lead to the formation of charges (positive and negative charges), the concentration of which is proportional to the concentration of electric dipoles. Assuming that the temporal evolution of electric dipoles follows the zero-order kinetics, we have the following:

(5) $$Q=Q_{0}+\alpha_{E}t$$

where Q and $Q_{0}$ are the charge concentrations at time *t* and *t* = 0, respectively, and α_E_ is the rate constant. For an RC circuit with the capacitance *C* = Q/V (V is voltage), the electric impedance, I, is as follows [23]:

(6) $$I=\sqrt{r^{2}+\frac{1}{\omega^{2}C^{2}}}$$

where r and ω are resistance and angular frequency, respectively. Using Equation (6), the normalized electric impedance is as follows:

(7) $$\frac{I}{I_{0}}=\frac{\sqrt{r^{2}+\frac{V^{2}}{\omega^{2}Q^{2}}}}{\sqrt{r^{2}+\frac{V^{2}}{\omega^{2}Q_{0}^{2}}}}≒1-\frac{\alpha_{E}t}{Q_{0}\omega^{2}C^{2}r^{2}}=1-b_{E}t$$

where $b_{E}$ is a constant proportional to the rate constant.

Using Equation (7) to fit the data in Figure 5, we obtain the numerical values of the fitting parameter, $\alpha_{E}$, for the animal oils at different temperatures. For comparison, the fitting curves are included in the corresponding plots in Figure 5. It is evident that Equation (7) accurately describes the temporal evolution of the electrical impedances of the animal oils. As discussed above, the temperature dependence of the rate constant follows the Arrhenius relation. The temperature dependence of the rate constant is shown in Figure 8c for the animal oils, from which the activation energies for the evolution of electric dipoles are found to be 44.2, 25.8, 39.2, and 36.4 kJ/mol for tallow, chicken oil, lard, and sheep oil, respectively. The livestock oils (tallow, lard, and sheep oil) have greater activation energies for the electric dipole than the poultry oil (chicken oil). Compared to the activation energies obtained from the absorbances and viscosities, we note that the activation energies for the evolution of electric dipoles are basically the same as the corresponding ones obtained from the evolution of color centers. Such a result suggests that the evolution of electric dipoles is correlated with the oxidation of triglycerides.

When an animal oil is exposed to the air or subjected to heating for a long time, it hydrolyzes progressively. Free fatty acid and glycerin are generated. A higher acid value means more free fatty acids in the oil, and the oil becomes spoiled. According to Sales-Campos et al. [27], fat deteriorates and is harmful to the human body when the acid value of cooking oil exceeds 3.0 mg KOH/g. From Figure 6, the time it takes for the acid value of the animal oils to reach 3.0 mg KOH/g, t_A_, increases with decreases in the heating temperature. In general, the reciprocal of the time to reach the acid value of 3.0 mg KOH/g can be expressed as follows:

(8) $$\frac{1}{t_{A}}=\frac{1}{t_{A0}}\exp\left(-Q_{\mathrm{AV}}/\mathrm{RT}\right)$$

where $Q_{\mathrm{AV}}$ is the activation energy for the hydrolyzation, $t_{A0}$ is a pre-exponential factor, R is the gas constant, and T is the absolute temperature.

Figure 8d shows the temperature dependence of $t_{A}^{1}$ for the animal oils. Using Equation (8) to fit the data in Figure 8d, the activation energies for the hydrolyzation are 41.5, 22.8, 37.9, and 37.5 kJ/mol for tallow, chicken oil, lard, and sheep oil, respectively. It is interesting to note that the activation energies for the hydrolyzation are nearly the same as the corresponding ones obtained from the measurements of electric impedance, dynamic viscosity, and UV-Vis absorbance. Such a result suggests that the hydrolyzation is likely correlated with the oxidation of triglycerides.

The thermal aging kinetics of various plant oils, including sunflower, soybean, olive, and canola oils, were investigated in our previous work [23]. The activation energies for the thermal degradation of these oils, determined from the measurements of absorbance, electrical impedance, and viscosity, were generally found to be lower than 30 kJ/mol. In contrast, the activation energies of the animal oils studied in this work are higher than 30 kJ/mol, with the exception of chicken oil. Such a difference in activation energy suggests that animal oils are less susceptible to reactions, such as oxidation, leading to thermal aging. Animal fats are primarily high in saturated fatty acids, which have no double bonds in their carbon chains, making them solid at room temperature, more chemically stable, and less prone to oxidation. In contrast, vegetable oils are rich in unsaturated fatty acids, which contain one or more double bonds; they are present in a liquid state at room temperature and have a greater susceptibility to oxidation and rancidity. The findings from the thermal aging kinetics of these two types of oil systems are consistent with the corresponding chemical properties.

According to Figure 7 and Figures S8–S11 in the Supplementary Information, the FTIR spectra of the animal oils are highly similar. The main peak near 3010 cm^−1^ is associated with the C–H stretching vibration of a cis-double bond (=CH); the two sharp peaks at 2923 and 2852 cm^−1^ represent the symmetric and asymmetric stretching vibration of the aliphatic CH_2_ group, respectively. The sharp peak at 1747 cm^−1^ represents the ester carbonyl functional group of triglycerides; a peak at 1740 cm^−1^ represents the C=O stretching aldehyde. A peak at 1465 cm^−1^ is associated with the bending vibrations of the CH_2_ and CH_3_ aliphatic groups; a small peak at 1378 cm^−1^ represents the bending vibrations of the CH_2_ groups. The peaks at 1239, 1162, and 1098 cm^−1^ are related to the stretching vibration of the C–O ester groups. A peak at 1117 cm^−1^ represents aliphatic esters. A peak at 722 cm^−1^ is related to the CH_2_ rocking vibration and the out-of-plane vibration of cis-disubstituted olefins. All of the peaks are the same as the corresponding ones reported by Vlachos et al. [28].

The changes in peak intensities are related to the changes in the fractions of unsaturated and saturated fatty acids during the heating. The decrease in the intensity peak at 1747 cm^−1^ is due to oxidation. A transformation from a C=C cis to C=C trans bond is observed in the FTIR spectra [29]. Notably, there is a significant difference in the contents of cis and trans fatty acids between sheep oil and the other oils, as shown in the FTIR spectra in the wavenumber range of 690–1050 cm^−1^ in Figure S12. The peaks at 722 cm^−1^ and 966 cm^−1^ correspond to the out-of-plane bending of the =C–H bond in the trans and cis configurations, respectively. The spectra reveal that sheep oil exhibits significantly weaker cis peaks and stronger trans peaks than the other three animal oils, indicating a higher fraction of trans fatty acids. With the increase in the heating time at a given temperature, the 966 cm^−1^ cis peak diminishes, while the 722 cm^−1^ trans peak intensifies, demonstrating the chemical reaction-induced conversion from a C=C cis to C=C trans bond.

## 4. Experimental Details

Four animal fats including tallow (Yellow Cattle), chicken (Aviagen), lard (LYD), and sheep (Nubian Goat) were bought from a local market in Hsinchu, Taiwan. All of the fats were stored at −3 °C in a 10.0LMF4-9B1X refrigerator (MicroFridge, Danby, Foxboro, MA, USA) for more than 3 days to prevent rotting prior to being used. Animal oils were extracted from corresponding fats by placing the fats in a 500 mL beaker, which was placed on a hot plate (KR-S2, Cadco, Winsted, CT, USA) at room temperature. The temperature of the hot plate was increased to a preset temperature of 100 °C after it was turned on, and maintained for half hour. The derived animal oils were sealed and stored in a dark cabinet at room temperature.

The aging temperature must be lower than the smoke point of the oil. The smoke points of tallow, chicken oil, lard, and sheep oil are 250, 190,185, and 190 °C, respectively [30,31]. The alternative name for sheep oil is mutton tallow. The measurements of the properties of the prepared animal oils were heat-treated at four different temperatures of 120, 140, 160, and 180 °C. Briefly, 125 mL of oil was poured into a 250 mL beaker, which was placed in a silicone oil bath to maintain a uniform temperature for different durations. A thermometer was used to measure the oil temperature to ensure a constant temperature over the heating period. The beaker with the oil was removed from the silicone oil bath after reaching the pre-determined time in the range from 0 to 900 h, and the oil temperature was cooled down to room temperature naturally. Each property measurement was repeated at least twice under the same conditions, and the results were compared to ensure the reproducibility of the results. Additionally, all instruments used were carefully calibrated according to the standard procedures suggested by the companies.

The temporal evolution of the transmittance of the animal oils in the wavelength range from 300 to 800 nm was recorded on a U-3900 ultraviolet–visible spectrophotometer (Hitachi High-Technologies Corporation, Tokyo, Japan). The transmittance of the blank was initially measured and used as the background, which was subtracted from the reported transmittances of the animal oils. At least two measurements were performed under the same conditions, and the results were compared to warrant the reproducibility of the experimental results. The intensity of absorbed light was maintained at ±0.005 au to limit errors. The dynamic viscosity of the animal oils was measured at room temperature on a Cannon-Ubbelohde 7990 S glass viscometer (Ramin, Magnolia, TX, USA). The sample size was 15 mL. The reported viscosity is the average value of five different measurements. The temporal evolution of the electrical impendence of the animal oils was determined on an LCR meter (8110G, Good Well Electronics Industrial Co., Ltd., New Taipei City, Taiwan) in the frequency range from 1000 Hz to 3000 Hz. using homemade interdigital sensors prepared using laser direct writing technology. The finger interspace of the interdigital sensor is 100 µm. The FTIR spectra of the animal oils were recorded on a Perkin Elmer Spectrum One FTIR (Perkin Elmer Inc., Waltham, MA, USA) for the wavenumber in the range from 500 to 3500 cm^−1^. The solutions used for the FTIR analyses consisted of KBr powder (99.9%, MSE Supplies, Tucson, AZ, USA) and the corresponding animal oils with a mass ratio of 100:1. At least two measurements were performed for the conditions, and the results were compared to warrant the reproducibility of the FTIR measurements.

A blank titration solution with 25 mL ethanol (MilliporeSigma, Darmstadt, Germany) and 25 mL diethyl ether (MilliporeSigma, Darmstadt, Germany) was prepared. In a beaker, a few drops of phenolphthalein (MilliporeSigma, Darmstadt, Germany) were added to the solution as an indicator, along with 5 g of animal oil and 50 mL of the blank titration, to form a mixture. The mixture was titrated with 0.01 M KOH (MilliporeSigma, Darmstadt, Germany) until the discoloration of the indicator. The titration of the blank titration solution was finished when the first stable pink of the same intensity appeared. The color is supposed to be maintained for 10 s.

## 5. Summary

In summary, we have studied the thermal aging kinetics of four different animal oils, including tallow, chicken oil, lard, and sheep oil, in the temperature range from 120 °C to 180 °C. Four different techniques were used in the characterization of the thermal aging of the animal oils, including the determinations of absorbance, dynamic viscosity, electric impedance, and acid titration. The following summarizes the main results obtained in this work.

1. The temporal evolution of the absorbance can be described by the second-order evolution of color centers.
2. The temporal evolution of the dynamic viscosity can be described by the first-order evolution of defects.
3. The electric impedance at 1000 Hz is a linearly decreasing function of the heating time.
4. Increasing the heating temperature accelerates the hydrolyzation of animal oils.
5. The activation energies determined independently by the temperature dependencies of the absorbance, dynamic viscosity, electric impedance, and acid titration are the same. Such a result indicates that the thermal aging kinetics of the animal oils are attributed to the oxidation of triglycerides.

These findings are relevant to the practical use of animal oils, as they provide critical insights into the stability of these oils under thermal conditions. Understanding the kinetics of thermal aging allows for the better prediction of how long these oils can maintain their quality during storage and processing. Additionally, the identified activation energies and the mechanisms behind property changes can inform the development of preservation strategies, such as optimizing storage temperatures and minimizing oxidative degradation, thereby enhancing the practical usability of animal oils in food, cosmetic, and industrial applications.

## Data Availability

Data are contained within the article and Supplementary Materials.

## Supplementary Materials

The following supporting information can be downloaded at: https://www.mdpi.com/article/10.3390/molecules29174191/s1, Figures S1–S3: Transmittance spectra of the animal oils at 160/140/120 °C for different durations: (a) tallow, (b) chicken oil, (c) lard, and (d) sheep oil. Figure S4: Variation of maximum difference of transmittances at the initial time and final time with wavelength at different temperatures: (a) tallow, (b) chicken oil, (c) lard, and (d) sheep oil. Figures S5–S7: Frequency dependence of electric impedances of the animal oils at 160/140/120 °C at different heating times: (a) tallow, (b) chicken oil, (c) lard, and (d) sheep oil. Figures S8–S11. FTIR spectra of tallow/chicken oil/lard/sheep oil with different heating durations at 120 °C: (a) overall, (b) 722 cm^−1^, (c) 1000–1500 cm^−1^, (d) 1744 cm^−1^, (e) 2800–3000 cm^−1^, and (f) 3008 cm^−1^. Figure S12. FTIR spectra in the wavenumber range of 690–1050 cm^−1^ of the four animal oils with different heating durations at 120 °C.
