# Supplementary material for: Analysis of the Thermal Aging Kinetics of Tallow, Chicken Oil, Lard, and Sheep Oil
Source: Molecules. 2024 Sep 4;29(17):4191. doi: 10.3390/molecules29174191 (PMC11396900; doi:10.3390/molecules29174191)
Supplement: Supplementary file 1 [file molecules-29-04191-s001.zip › molecules-3151221-supplementary.pdf]

Analysis of the Thermal Aging Kinetics of Tallow, Chicken Oil, Lard, and Sheep Oil

Yun-Chuan Hsieh [1)], Hao Ouyang [1)], Yulin Zhang [2)], Donyau Chiang [3)], Fuqian Yang [4)], Hsin-Lung Chen [5)], Sanboh Lee [1),*]

1) Department of Materials Science and Engineering, National Tsing Hua University, Hsinchu 300, Taiwan
2) Chongqing Institute of Green and Intelligent Technology, Chinese Academy of Sciences, Chongqing 400714, China
3) National Applied Research Laboratories, Taiwan Instrument Research Institute, Hsinchu 300, Taiwan
4) Materials Program, Department of Chemical and Materials Engineering, University of Kentucky, Lexington, KY 40506, USA
5) Department of Chemical Engineering, National Tsing Hua University, Hsinchu 300, Taiwan

[*] Corresponding author. Email: sblee@mx.nthu.edu.tw; Telephone/Fax: +88635719677

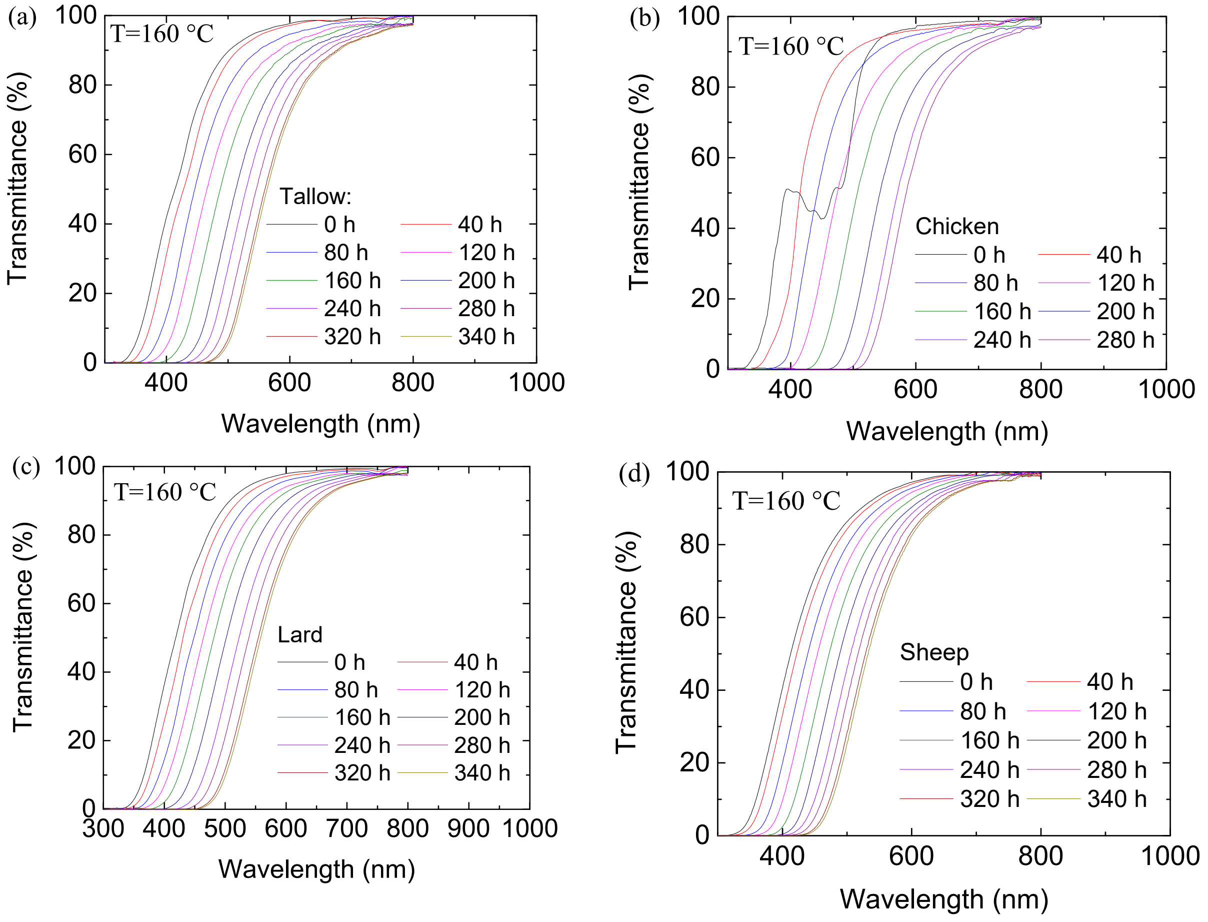

Figure S1. Transmittance spectra of the animal oils at 160 ºC for different durations: (**a**) tallow, (**b**) chicken oil, (**c**) lard, and (**d**) sheep oil.

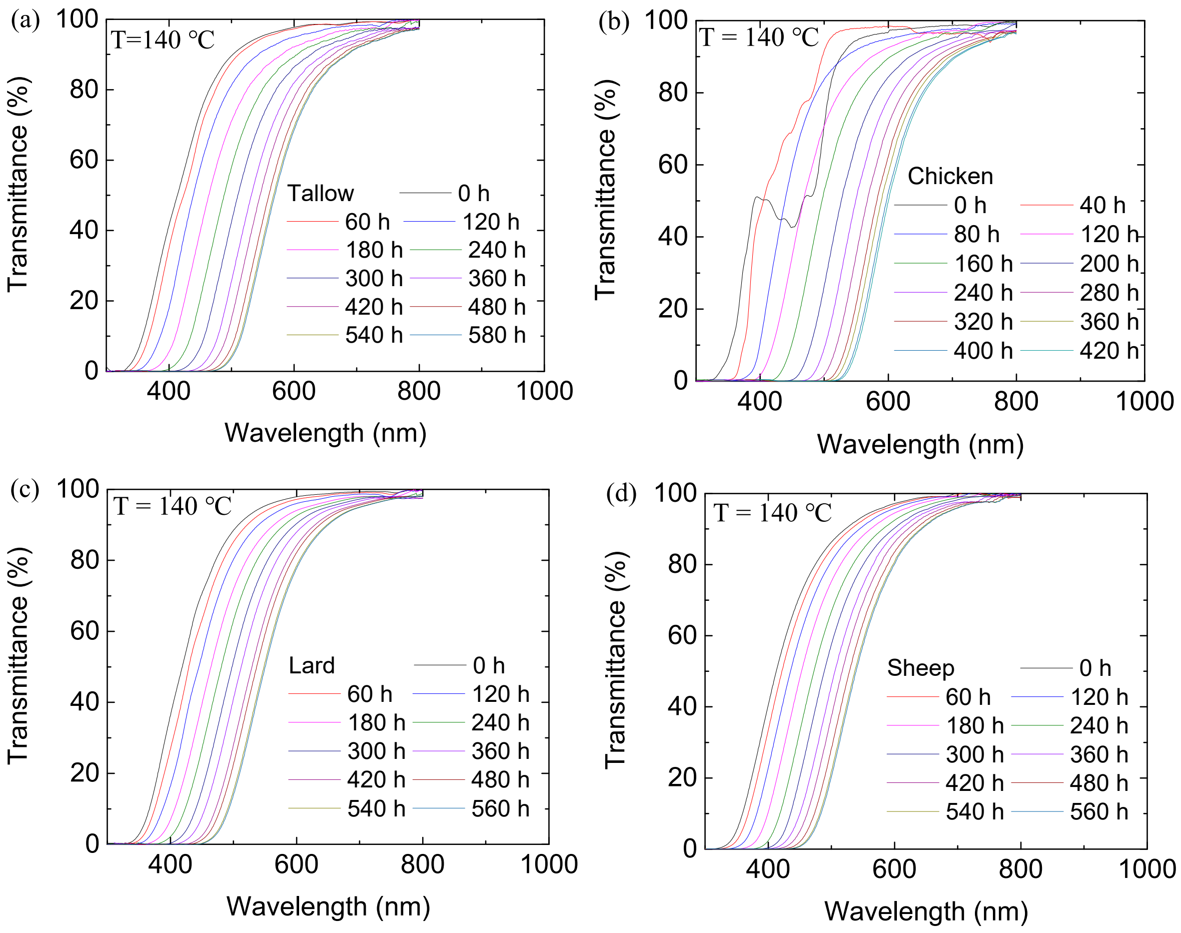

Figure S2. Transmittance spectra of the animal oils at 140 ºC for different durations: (**a**) tallow, (**b**) chicken oil, (**c**) lard, and (**d**) sheep oil.

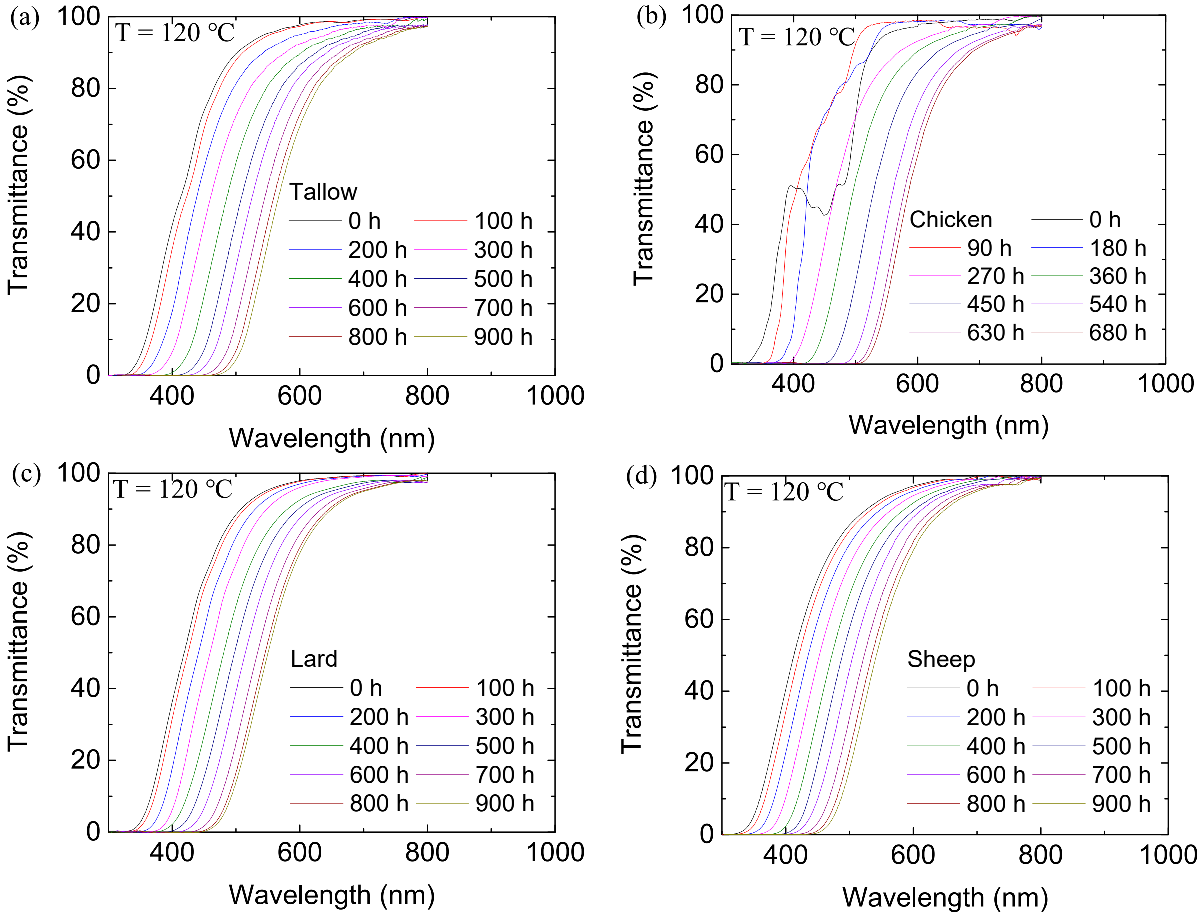

Figure S3. Transmittance spectra of the animal oils at 120 ºC for different durations: (**a**) tallow, (**b**) chicken oil, (**c**) lard, and (**d**) sheep oil.

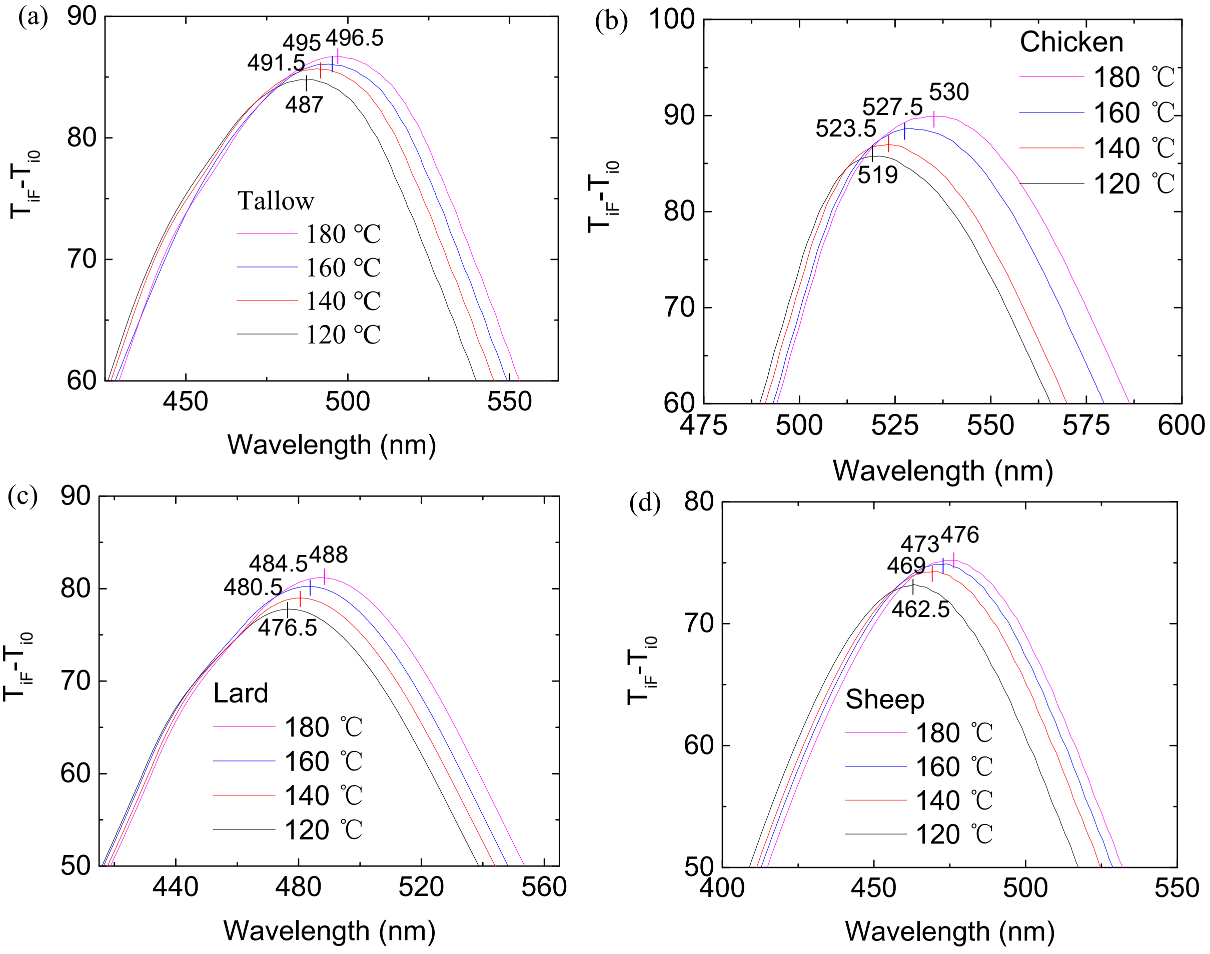

Figure S4. Variation of maximum difference of transmittances at the initial time and final time with wavelength at different temperatures: (**a**) tallow, (**b**) chicken oil, (**c**) lard, and (**d**) sheep oil.

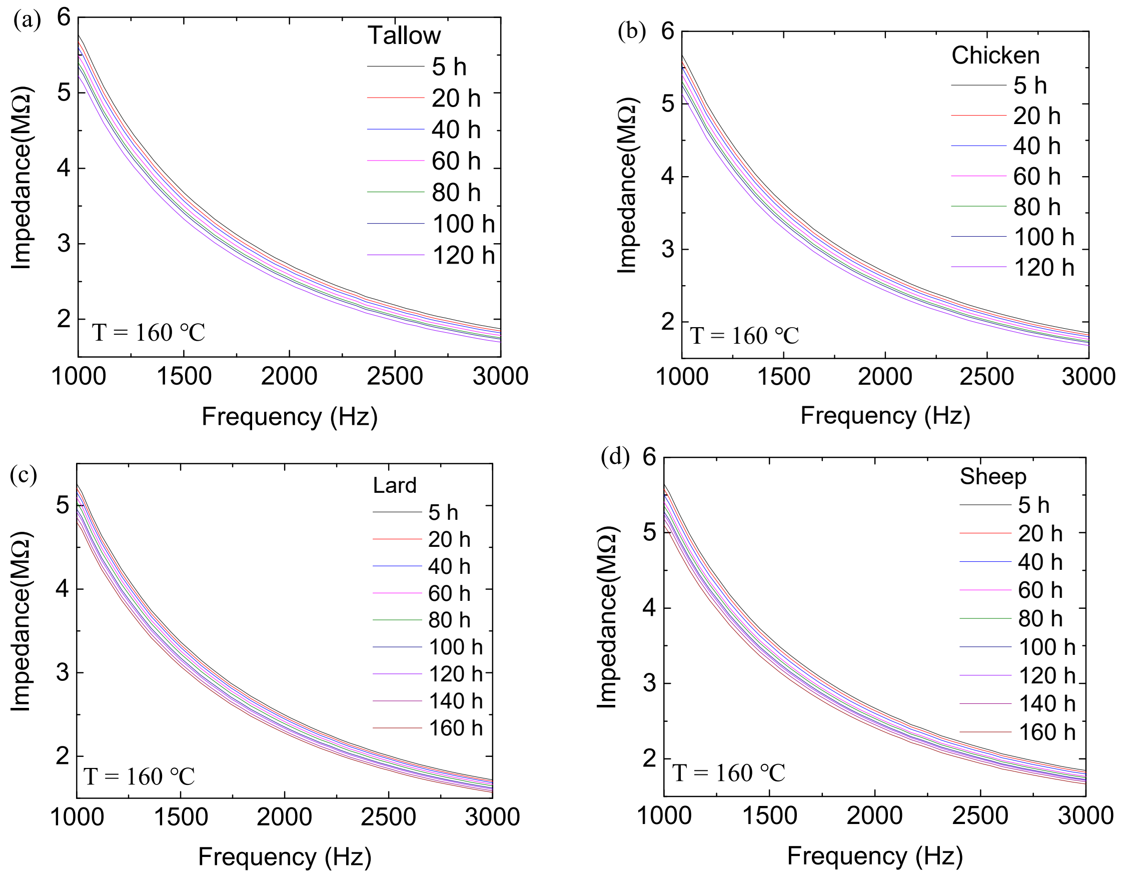

Figure S5. Frequency dependence of electric impedances of the animal oils at 160 °C at different heating times: (**a**) tallow, (**b**) chicken oil, (**c**) lard and (**d**) sheep oil.

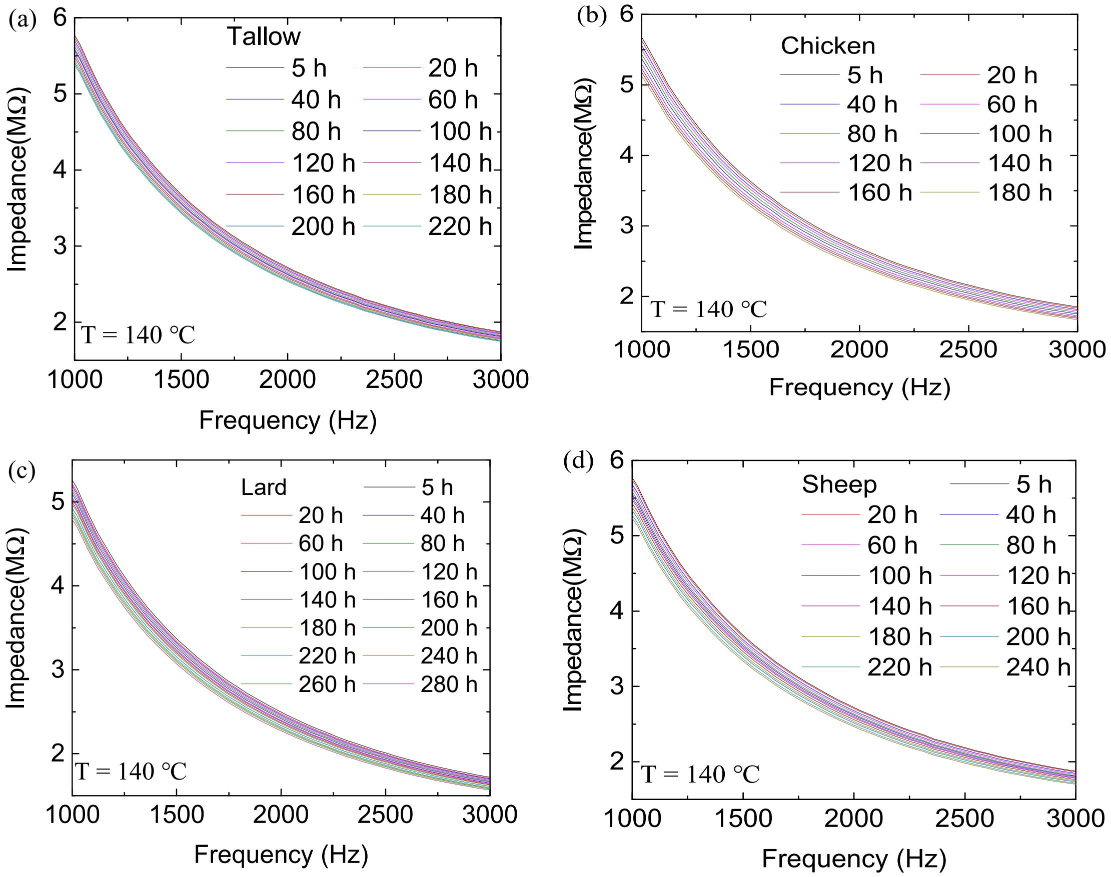

Figure S6. Frequency dependence of electric impedances of the animal oils at 140 °C at different heating times: (**a**) tallow, (**b**) chicken oil, (**c**) lard, and (**d**) sheep oil.

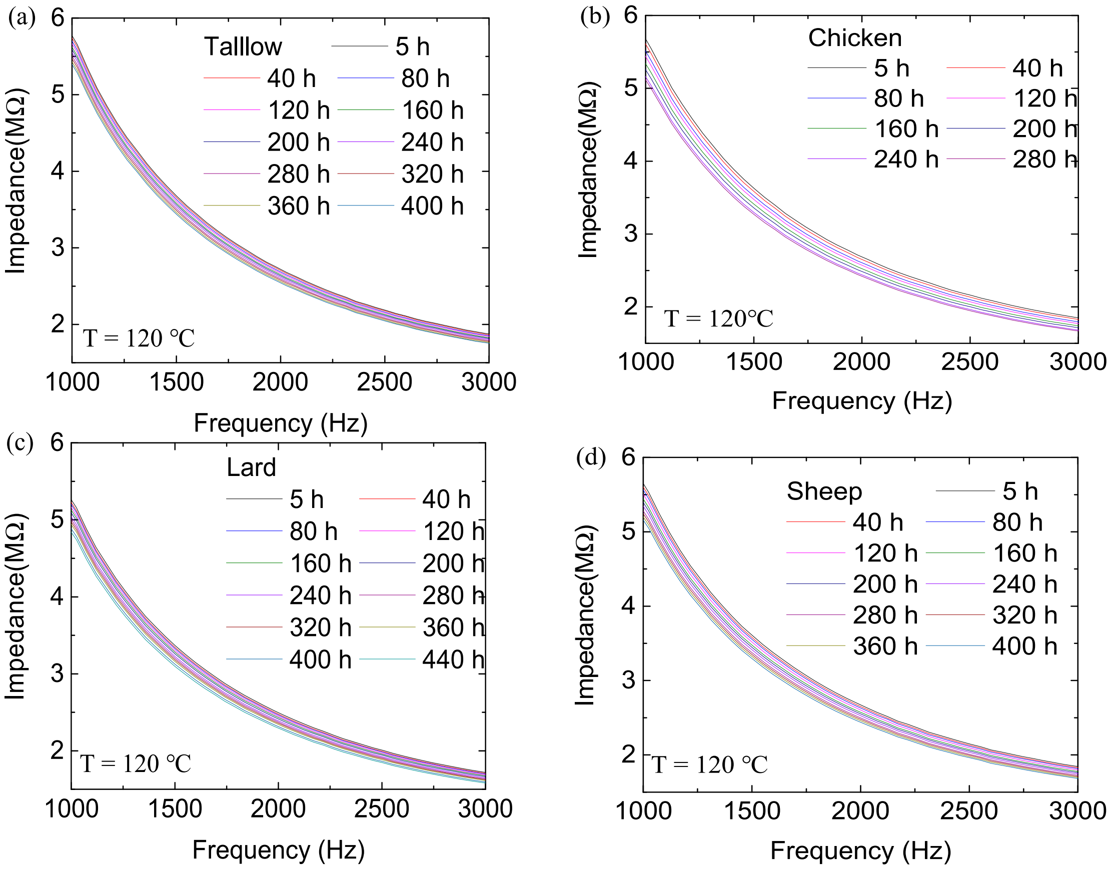

Figure S7. Frequency dependence of electric impedances of the animal oils at 120 °C at different heating times: (**a**) tallow, (**b**) chicken oil, (**c**) lard, and (**d**) sheep oil.

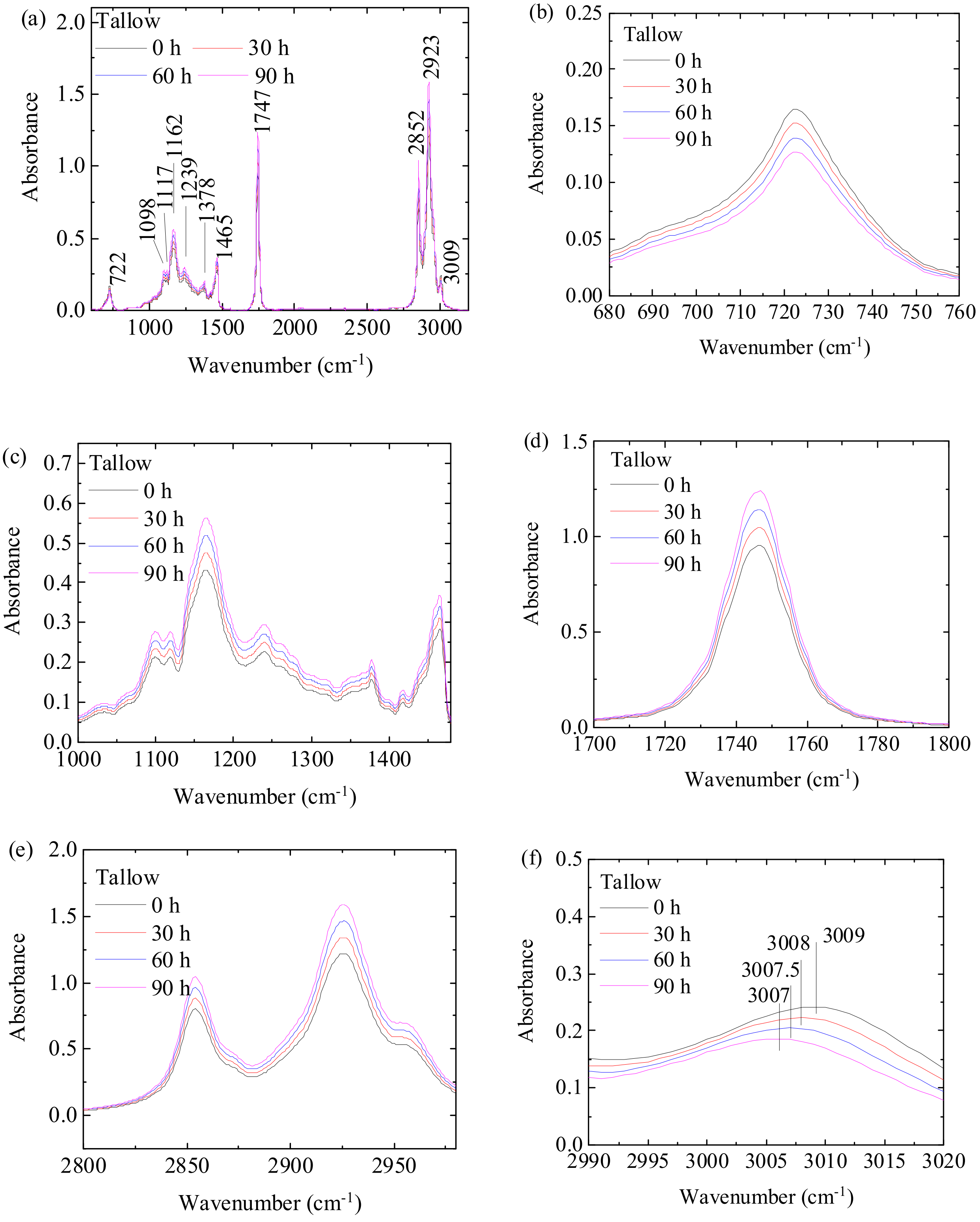

Figure S8. FTIR spectra of tallow with different heating durations at 120 °C: (**a**) overall, (**b**) 722 cm^{-1}, (**c**) 1000-1500 cm^{-1}, (**d**) 1744 cm^{-1}, (**e**) 2800-3000 cm^{-1}, and (**f**) 3008 cm^{-1}.

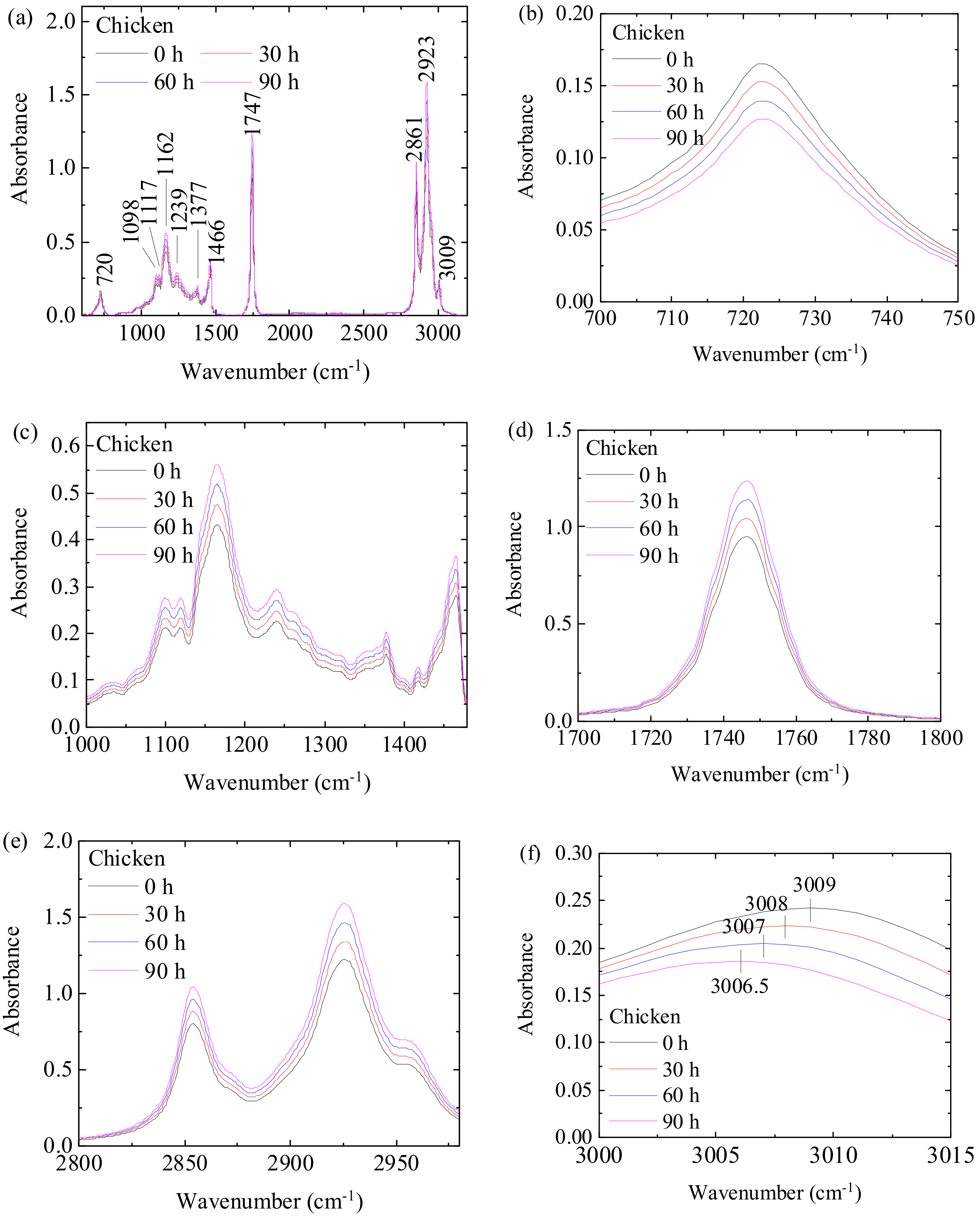

Figure S9. FTIR spectra of chicken oil with different heating durations at 120 °C: (**a**) overall, (**b**) 722 cm^{-1}, (**c**) 1000-1500 cm^{-1}, (**d**) 1744 cm^{-1}, (**e**) 2800-3000 cm^{-1} and (**f**) 3008 cm^{-1}.

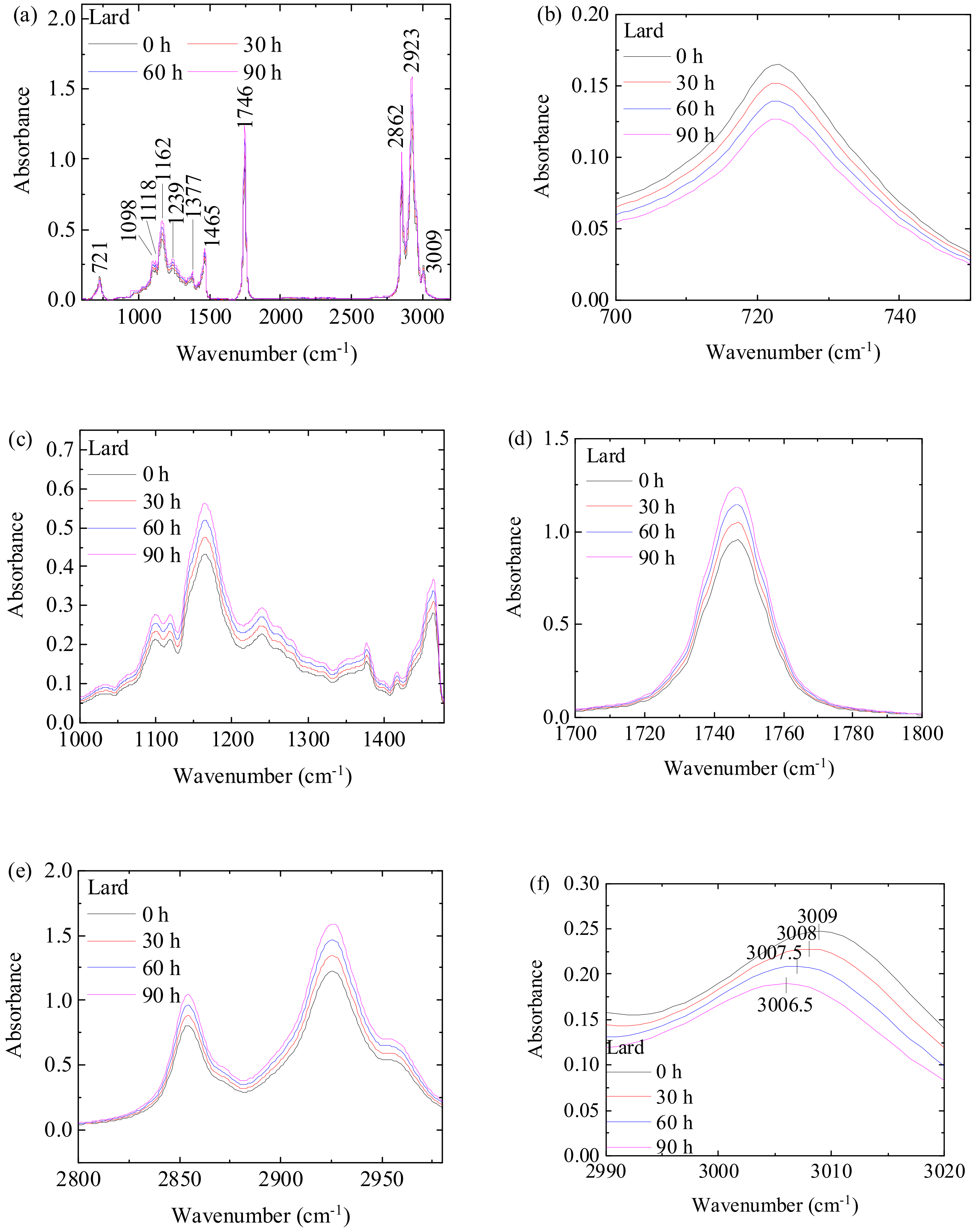

Figure S10. FTIR spectra of lard with different heating durations at 120 °C: (**a**) overall, (**b**) 722 cm^{-1}, (**c**) 1000-1500 cm^{-1}, (**d**) 1744 cm^{-1}, (**e**) 2800-3000 cm^{-1}, and (**f**) 3008 cm^{-1}.

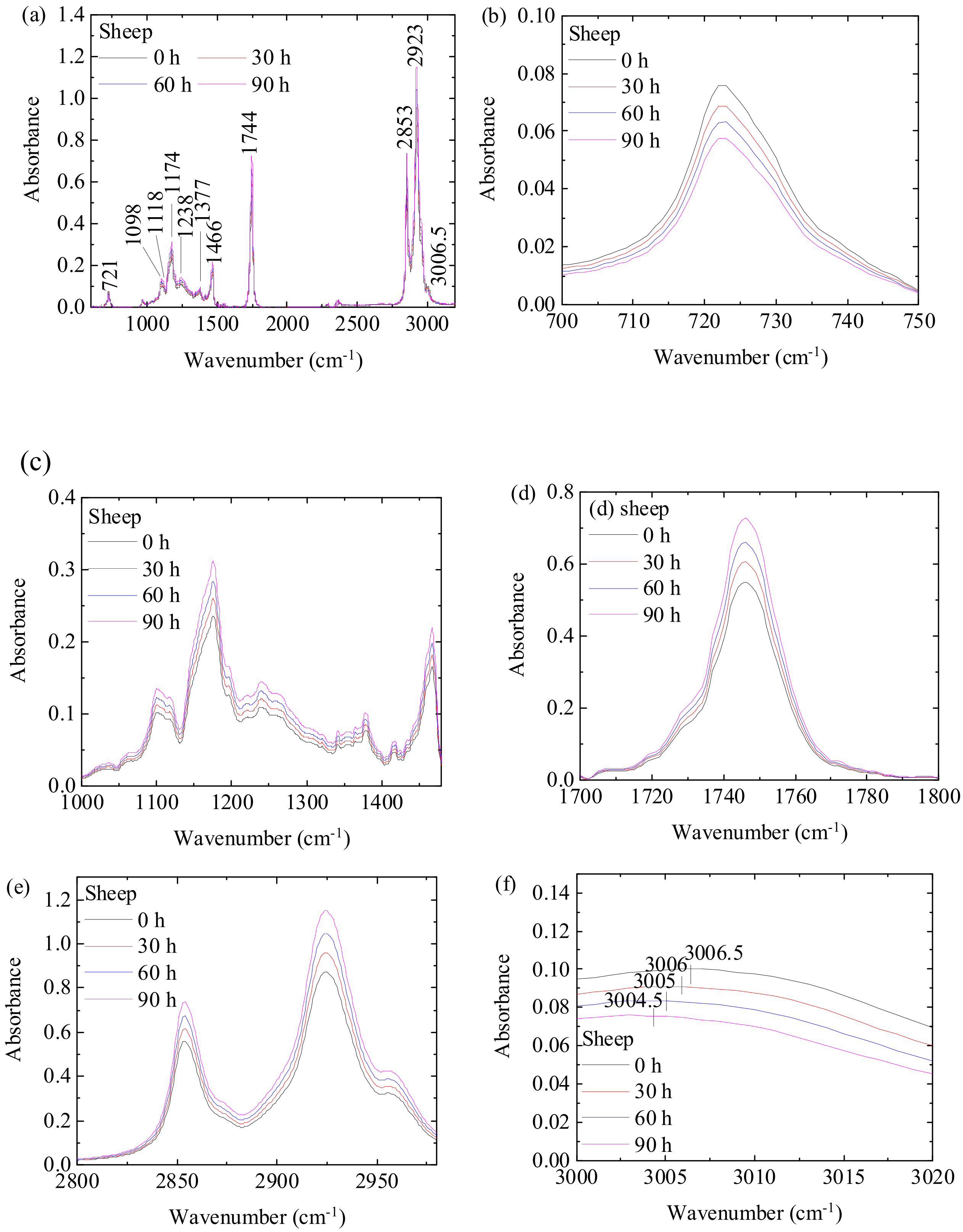

Figure S11. FTIR spectra of sheep oil with different heating durations at 120 °C: (**a**) overall, (**b**) 722 cm^{-1}, (**c**) 1000-1500 cm^{-1}, (**d**) 1744 cm^{-1}, (**e**) 2800-3000 cm^{-1}, and (**f**) 3008 cm^{-1}.

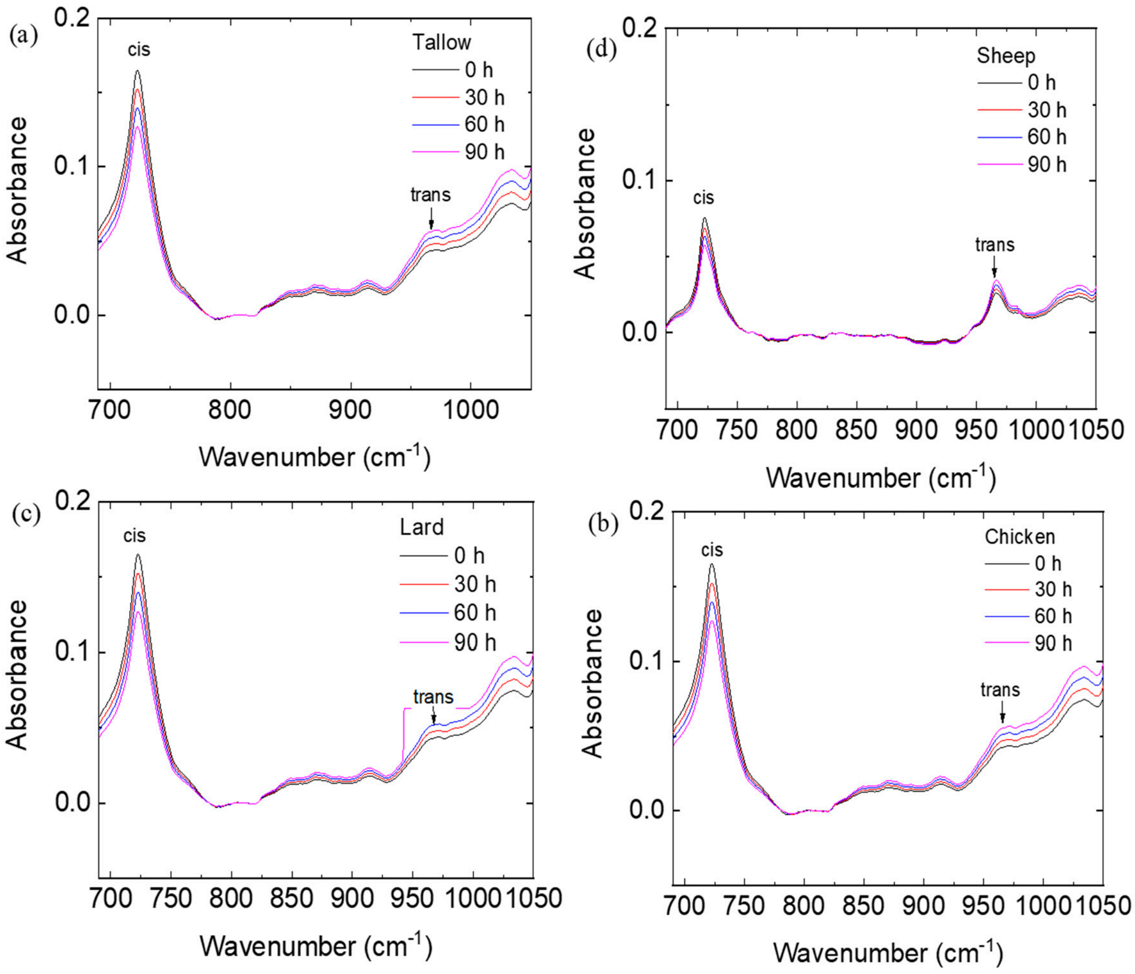

Figure S12. FTIR spectra in the wavenumber range of 690-1050 cm^{-1} of the four animal oils with different heating durations at 120 °C.
